# Genome-wide association study reveals candidate genes for litter size and function validation of *MMP16* gene in lop sheep

**DOI:** 10.3389/fvets.2025.1682236

**Published:** 2025-10-30

**Authors:** Jieru Wang, Lanshu Bi, Hong chen, Xiaopeng Li, Abliz Khamili, Waili Kurban, Chenchen Yang, Peng Niu, Fei Huang, Di Fang, Chunmei Han, Qinghua Gao

**Affiliations:** ^1^College of Animal Science and Technology, Tarim University, Alar, China; ^2^Xinjiang Bazhou Animal Husbandry Work Station, Bazhou, China; ^3^Bureau of Agriculture and Rural Development, Ruoqiang County, Bazhou, China; ^4^Agricultural Development Centre, Ruoqiang County, Bazhou, China; ^5^Key Laboratory of Livestock and Forage Resources Utilization Around Tarim, Ministry of Agriculture and Rural Areas, Alar, China

**Keywords:** lob sheep, whole-genome resequencing, litter size, MMP16, ovarian granulosa cells

## Abstract

As a premium indigenous sheep breed endemic to Tarim Basin, Xinjiang, China, Lop sheep demonstrate remarkable adaptability to extreme arid, high-temperature and sandstorm-prone environments, with some ewes exhibiting exceptional prolificacy. To elucidate the genetic regulation of this reproductive trait, we integrated whole-genome resequencing and transcriptome sequencing, conducting genome-wide association study on 110 Lop ewes with particular emphasis on the regulatory role in litter size determination of *MMP16* gene. Through multi-dimensional investigations including qRT-PCR, immunohistochemistry, ovarian granulosa cells culture, and transcriptome sequencing, we revealed that *MMP16* gene significantly influences folliculogenesis and ovulation by modulating extracellular matrix (ECM) remodeling and PI3K-AKT signaling pathway activation. Our research systematically elucidates genetic variations associated with prolificacy in Lop sheep and deciphers the biological function of *MMP16* gene, providing novel insights into the genetic architecture of ovine reproductive traits. The findings establish a theoretical foundation for molecular marker-assisted breeding and the exploitation of superior germplasm resources.

## 1 Introduction

Sheep constitute a cornerstone of global livestock production systems, and their reproductive performance serves as the primary constraint that directly affects production efficiency and economic outcomes of the industry (1). Litter size is a critical reproductive trait in sheep (2–4). Enhancing this trait can significantly increase the number of sheep (5–7), enabling sustainable provision of high-quality lamb, wool, and dairy products to meet growing market demands. Lop sheep, a superior indigenous breed originating from the Tarim Basin, exhibits exceptional adaptation to extreme climatic conditions, robust genetic stability, and pronounced disease resistance. Therefore, investigating the genetic mechanisms underlying their fecundity is essential for refining breeding programs and advancing ovine reproductive efficiency.

Several important genes influencing sheep litter size have been identified, such as *BMPR1B*, *BMP15*, and *GDF9*, which can regulate the synthesis and secretion of hormones related to follicular growth and ovulation in sheep (8, 9). However, litter size in different sheep breeds is regulated by distinct major genes. For example, a mutation in the *PIK3CD* gene influences the litter size of Small Tail Han sheep by affecting its expression level (10), while a 24-bp indel in the *AHR* gene is associated with litter size and the number of live lambs in Australian White sheep (11). Genetic studies of sheep litter size have concentrated on the biological mechanisms underlying follicular development and ovulation, particularly how key genes modulate dominant follicle selection and ovulation rate, suggesting that follicular development directly influences ewe lambing potential.

In female mammals, follicular development is a key component of the reproductive cycle, whereby the number of mature follicles directly dictates the ovulation rate (12). Ovarian granulosa cells (GCs) surround oocytes to form follicular structures, which play pivotal roles in regulating follicular growth, hormone secretion, oocyte maturation, and embryonic development (13–15), and their proliferation and differentiation capacities directly determine the growth potential of follicles (16, 17). Additionally, apoptosis of GCs directly induces follicular atresia (18), which decreases ovulation rate and thereby directly impacts litter size. Thus, ovarian GCs serve as an ideal *in vitro* model for investigating sheep fecundity.

Traditional breeding methods exhibit a long cycle and high variability in outcomes (19), whereas molecular marker-assisted breeding techniques enable precise screening of genotypes associated with litter size control at the DNA molecular level, improving the accuracy of selection and shortening the reproductive cycle (20, 21). Recent advancements in biotechnology have propelled high-throughput sequencing technology to become an indispensable tool for discovering superior traits in genetics research. It not only reveals the genetic basis of sheep fecundity but also provides new molecular markers and breeding strategies for sheep breeding (6, 22–24). Chantepie identified SNPs significantly associated with sheep litter size using ovine 50 k SNP chip (25). Zhang obtained key genes related to sheep tail type and wool quality traits through joint analysis of genome-wide association study (GWAS) and selective sweep (26). Pokharel performed integrated mRNA-miRNA analysis to identify candidate genes associated with litter size in Finn sheep and Texel sheep (27). Additionally, as the cost of whole genome sequencing, the gold standard for GWAS, continues to decrease (28–30), GWAS has become increasingly widely applied in sheep populations. Xiang reveals five new genes (*MAP3K1*, *ANKRD55*, *ABCB1*, *MEF2C*, and *TRNAW*-*CCA*-*87*) related to growth hormone and energy metabolism that are significantly associated with sheep body weight traits through whole genome sequencing and GWAS analysis (31). By capitalizing on its strengths in genome-wide coverage, robust statistical power, and data-driven methodologies, genome-wide association studies (GWAS) have significantly advanced the detailed dissection of the polygenic genetic architecture underlying complex traits (32–36).

In southern Xinjiang, China, we identified two Lop sheep populations characterized by high litter size and lamb survival rate, and proceeded to conduct the following studies using these populations as the research subjects: (1) Resequenced 110 Lop sheep and identified genetic variants significantly associated with litter size through GWAS. (2) Analyzed the expression profiles of the *MMP16* gene in different tissues, follicular cells, and performed ovarian spatiotemporal localization analysis to explore its role in follicular development. (3) Conducted the vitro model of overexpressing and interfering with *MMP16* in GCs to investigate its effects on follicular development. Our study reveals the key regulatory role of the *MMP16* gene in the formation of high fecundity traits in Lop sheep through the integration of population genetics, gene function validation, and molecular regulatory mechanism analysis. The findings have not only provided a theoretical basis for deciphering the genetic mechanisms underlying high fecundity in sheep but also laid a scientific foundation for developing molecular marker based breeding strategies for elite breeds.

## 2 Materials and methods

### 2.1 Animal care

Animal experiments were approved by the Animal Ethics Committee of the College of Animal Science and Technology of Tarim University (No. DTU 20230126).

### 2.2 Animal samples and tissues collection

Two populations of Lop sheep were sourced from agricultural facilities in Weili and Ruoqiang county, Xinjiang, China. All subjects were maintained under standardized husbandry conditions, receiving identical nutritional regimens and environmental parameters. No significant intergroup differences were observed in phenotypic metrics including mean age, body mass, somatometric measurements (such as body length, chest circumference), or other physiological characteristics. Whole blood samples were collected via jugular venipuncture using 5 mL K2EDTA anticoagulant tubes. Collected specimens were promptly aliquoted and preserved at −20 °C for subsequent analysis.

The sheep were divided into the single-lamb group (*n* = 3) and the multi-lamb group (*n* = 3) according to the record of litter size for three consecutive years. After estrus synchronization, sheep in the same physiological state underwent deep anesthesia through intravenous injection of 3% pentobarbital sodium solution (Solarbio, Beijing, China). Postmortem, reproductive neuroendocrine tissues (hypothalamus, pituitary) and reproductive tissues (ovary, uterus, fallopian tube) were surgically excised under aseptic conditions. Tissue specimens were dissected into 50–100 mg fragments using sterile surgical instruments, and flash-frozen in liquid nitrogen within 2 min of excision to preserve RNA integrity.

Fresh ovine ovaries were aseptically harvested and transported in phosphate-buffered saline pre-warmed to 37 °C, supplemented with antibiotic supplementation (100 IU/mL penicillin and 50 mg/mL streptomycin), and maintained at physiological osmolarity.

### 2.3 DNA extraction and genotyping

Genomic DNA was extracted from blood samples using the magnetic bead method according to the manufacturer’s instructions for the Blood DNA extraction Kit (TIANGEN, Beijing, China). The integrity of the extracted DNA was detected by 1% agarose gel, and the concentration of the DNA was determined using NanoDrop 2000 spectrophotometer (Thermo Fisher Scientific, Waltham, United States). The qualified DNA was submitted to Beijing Novogene Co., Ltd. for 10 × whole-genome resequencing.

The TruSeq DNA PCR-Free kit was used to construct a whole-genome sequencing library with an insertion fragment size of approximately 350 bp. The libraries were sequenced on the Illumina HiSeq 2000 platform, with each fragment generating a paired end read of 100 bp. FastQC software^1^ was used to evaluate the quality of the sequencing data. Fastp software was used for quality control of sequencing data (37), using default parameters. BWA-MEM algorithm was used to compare the sequencing data with the sheep reference genome (UI_Ramb_v2.0/GCF_016772045.1). The MarkDuplicates module in Picard Tools software was used to remove PCR duplicates. GATK 4.0 module HaplotypeCaller was used for mutation identification. Plink was used for quality control, and the single nucleotide polymorphisms (SNPs) were removed if the loss rate was less than 90%, the minor allele frequency was lower than 0.05, or the Hardy–Weinberg equilibrium was less than <10^−6^.

### 2.4 Genome-wide association studies

The linear mixed model is widely used in genome-wide association study because it can correct the population structure and the complex relationships within the population. The model is as follows:


y=Wα+Xβ+Zμ+e

where y is the litter size, α includes vectors of fixed effects (parity, years, and seasons), β is the vector of random effects, μ is the vector of permanent environmental effects, e is the vector of residual effects, and W, X, and Z are the correspondent incidence matrices of α, β, and μ, respectively.

### 2.5 Total RNA extraction, cDNA synthesis and quantitative real-time polymerase chain reaction

Total RNA was isolated using TRIzol reagent (Invitrogen, Carlsbad, United States) and the concentration of RNA were assessed by measuring absorbance at 260 nm and 280 nm using a spectrophotometer (Thermo, Waltham, MA, United States). Additionally, the integrity of RNA was assessed through 1.5% agarose gel electrophoresis. The cDNA was synthesized using a PrimeScript RT reagent kit (TaKaRa, Beijing, China). qPCR was performed using ChamQ Universal SYBR qPCR MasterMix (Vazyme, Nanjing, China). According to the manufacturer’s instructions, the amplification was carried out in a reaction system of 15 μL, and primer information was shown in Table 1.

**Table 1:** Primer information.

Gene	Primer sequence (5′–3′)	Annealing temperature (°C)	Product length (bp)
*MMP16*	F: TCAAGGGGGACAGGTATTGG	58	247
	R: GTTGGTCCATCACAGCCCAT		
*CCND1*	F: AACTACCTGGACCGCTTCCT	60	140
	R: TCGGTGTAGATGCACAGCTT		
*CDK2*	F: AACAAGTTGACGGGAGAAG	60	237
	R: AAGAGGAATGCCAGTGAGT		
*BAX*	F: TTCCGACGGCAACTTCAACT	58	127
	R: GTCCAATGTCCAGCCCATGA		
*BCL2*	F: TCATGTGTGTGGAGAGCGTC	60	98
	R: CTAGGGCCATACAGCTCCAC		
*CYP11A1*	F: ACCAGGTCCCAGCTACTTTC	58	148
	R: TCATGCATGCCGATGAACTG		
*STAR*	F: GCGACCAAGAGCTTGCCTAT	58	128
	R: TTTACTCAGCACCTCGTCCC		
*GAPDH*	F: GTTTGTGATGGGCGTGAACC	55	154
	R: GCGTGGACAGTGGTCATAAGT		

### 2.6 Immunohistochemistry

Ovine ovarian tissues were fixed by immersion in 4% paraformaldehyde for 24 h, progressively dehydrated through a graded ethanol series, embedded in paraffin blocks, and sectioned into 5 μm-thick slices. Sections were mounted on glass slides for subsequent histological processing. Tissue sections were deparaffinized, rehydrated through a graded ethanol series, and washed with PBS three times for 5 min each. Following PBS washing, tissue sections were probed with rabbit anti-MMP16 primary antibody (Affinity Biosciences, Changzhou, China), horizontally positioned in a humidity-controlled incubation chamber, and maintained at 4 °C for 12–16 h. Secondary antibodies were applied and incubated at room temperature for 10 min. Nuclei were counterstained with hematoxylin (2–3 min), rinsed under running tap water for chromatin visualization, dehydrated through ascending ethanol concentrations and cleared in xylene.

### 2.7 Isolation and culture of sheep ovarian granulosa cells

Ovaries were sequentially washed three times with sterile saline solution containing antibiotics (100 U/mL penicillin, 50 μg/mL streptomycin), with 1–2 min cycles per wash. Following superficial adipose tissue excision with aseptic forceps, specimens were processed through two subsequent sterile saline rinsing cycles under laminar airflow conditions. Ovarian follicular dissection was conducted under aseptic conditions within a sterile culture dish employing microsurgical scalpels, utilizing controlled digital pressure to facilitate intact follicular antrum evacuation. The aspirated follicular fluid was transferred to 15 mL conical centrifuge tubes and subsequently subjected to centrifugation at 1,500 g for 5 min. Supernatant was carefully decanted, retaining the granulosa cell-enriched pellet. Pelleted cells were resuspended in 1 mL complete growth medium (DMEM supplemented with 10% FBS). Cultures were maintained at 37 °C in a humidified 5% CO₂ incubator for subsequent expansion.

### 2.8 Cell transfection

Ovine GCs were plated in 6-well culture plates containing 2 mL of DMEM (Gibco, New York, United States) per well, and maintained at 37 °C in a humidified 5% CO_2_ atmosphere. When the cells reached 70–80% confluence, the original culture medium was discarded, and the cells were washed twice with 1 mL of PBS. The cells in each well were transfected with 100 pmol of siRNA or 4 μg lenti-*MMP16* according to the manufacturer’s protocol for Lipofectamine 2000 (Invitrogen, Carlsbad, United States).

### 2.9 Cell proliferation test

Inoculated GCs into 96-well plates. After transfection, 10% CCK-8 solution was added to each well in accordance with the CCK-8 instructions. After culturing in the cell culture incubator, the absorbance at 450 nm was determined using an microplate reader.

EdU incorporation was analyzed using the EdU Cell Proliferation Kit (RiboBio, Guangzhou, China) following the manufacturer’s standardized protocol. According to the manufacturer’s instructions, cells were sequentially processed as follows: initial incubation with EdU solution for 2 h at 37 °C, followed by two washes with PBS. Subsequently, cells were fixed with 4% paraformaldehyde in PBS for 30 min at room temperature. After fixation, samples underwent two additional PBS washes. Permeabilization was then performed using 0.5% Triton X-100 solution (100 μL per sample) for 10 min at ambient temperature. Add 100 μL of staining reaction solution to each well and incubate at room temperature on a shaker for 30 min. Then add 100 μL reaction solution to each well and incubate at room temperature for 30 min. Finally, wash three times with PBS and place the cells under a fluorescence microscope for observation.

### 2.10 TUNEL

Fixed cells were gently aspirated and subjected to a single PBS rinse. Add PBS containing 0.3% Triton X-100 (Beyotime, Shanghai, China) to cells and incubate at room temperature for 5 min. Wash twice with PBS, add 100 μL of TUNEL (TdT enzyme and fluorescent labeling solution) detection solution, and incubate for 60 min. Wash with PBS three times, seal the slides and observe under a fluorescence microscope. Post-incubation specimens underwent three PBS washing cycles, mounted under coverslips using antifade medium, and imaged via laser scanning confocal microscopy with standardized emission settings.

### 2.11 Protein extraction and western blot

The pelleted cell was loaded onto a spin column, and 200 μL cell lysis buffer was added. After incubation at room temperature for 2 min, the mixture was centrifuged at 16,000 r/min for 2 min. The flow-through was immediately placed on ice, and the spin column was discarded to complete protein extraction. Protein concentrations were measured and adjusted to equal levels, followed by denaturation in boiling water. The 12% separating gel and 5% stacking gel were prepared using an SDS-PAGE gel kit for protein electrophoresis. Following SDS-PAGE, the gel region containing the target protein was excised and transferred onto a PVDF membrane. The membrane was blocked with blocking buffer for 60 min, then incubated with primary antibody (Affinity biosciences, Cincinnati, United States) at room temperature for 60 min. Subsequently, secondary antibody (Epizyme, Shanghai, China) was applied and incubated at room temperature for 60 min, with GAPDH serving as the internal control protein and imaging was performed using a ChemiDoc chemiluminescence detection system.

### 2.12 Transcriptome sequencing

After overexpressing the *MMP16* gene in GCs, transcriptome sequencing was performed. Using CASAVA base calling, the image data of sequencing fragments obtained from high-throughput sequencers were converted into sequence data in FASTQ format. These files underwent filtration to remove raw data, sequencing error rate verification, and GC content distribution evaluation, ultimately yielding clean reads for subsequent analysis. The clean reads were aligned to the reference genome using HISAT2 software to rapidly and accurately obtain read localization information on the reference genome (38). Quantitative analysis was conducted using the featureCounts tool from the subread software package (39). Subsequently, statistical analysis of expression data was performed to identify genes with significantly different expression levels across sample conditions. The clusterProfiler software was employed for Kyoto Encyclopedia of Genes and Genomes (KEGG) pathway enrichment analysis of differentially expressed genes (DEGs), aiming to identify major affected biological functions or pathways (40).

### 2.13 Statistical analysis

All experimental procedures were performed in triplicate biological replicates, and cycle threshold (CT) values were calculated using the 2^−ΔΔct^ method. SPSS 24.0 was used to analyze whether the difference was significant by one-way ANOVA analyses. The difference was considered significant at *p* < 0.05, and * represents *p* < 0.05, ** represents *p* < 0.01 and ns represents no significant difference.

## 3 Results

### 3.1 Sequencing quality analysis and quality control

Whole-genome resequencing was conducted on 110 Lop sheep, yielding a total raw sequencing data output of 5,236.34 Gb. The sequencing depth ranged from a minimum of 4.32 × to a maximum of 11.55×, with an average depth of 7.45 × and an average coverage of 98.20% (Q20 ≥ 98.17%, Q30 ≥ 96.71%). The alignment rate to the reference genome exceeded 99.4%. Variants were annotated primarily to intergenic regions of coding genes, totaling 8,497,913, followed by intronic regions of coding genes, with 4,875,735 variants. After quality control, 37,772,316 SNPs were retained for further analysis.

### 3.2 Genome-wide association study

**Figure 1:**
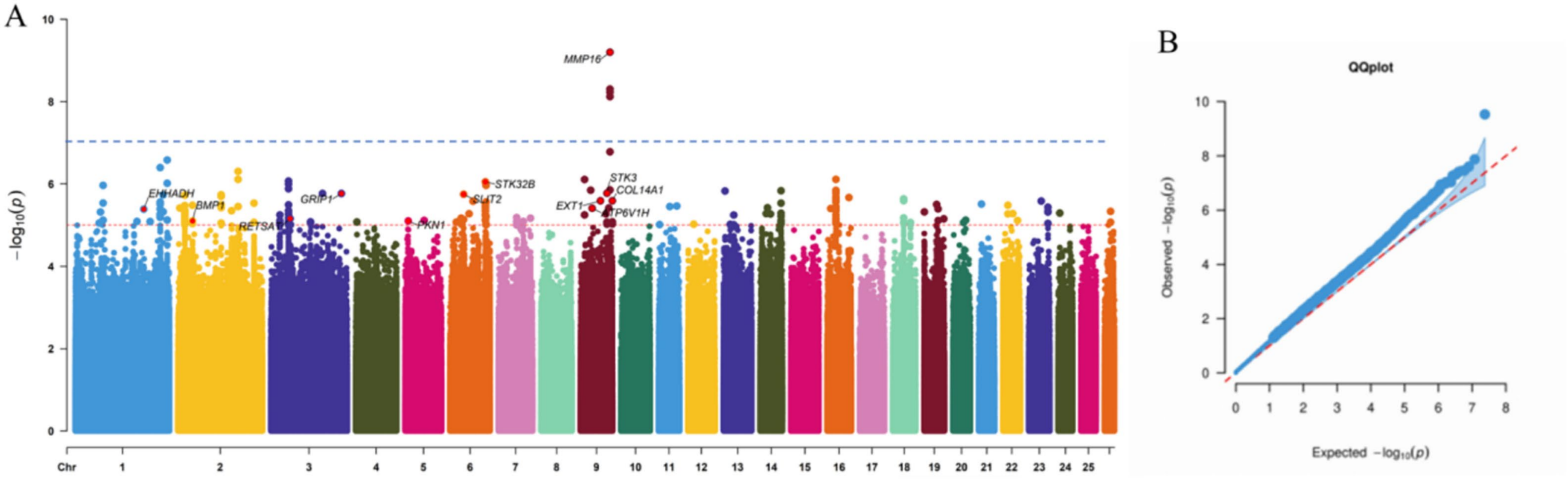
Manhattan and quantile-quantile plot of SNP GWAS. **(a)** The blue line corresponds to the 5% chromosome-wide significance threshold using a Bonferroni correction (10^−7^). The rede line corresponds to a suggestive chromosome-wide threshold of 10^−5^. **(b)** Quantile-quantile plot of GWAS.

To identify genetic variants associated with reproductive traits in Lop sheep, we conducted GWAS using genotype data from 110 individuals and phenotypic records of litter size. The Manhattan plot revealed multiple candidate loci associated with litter size, including genes such as *MMP16*, *BMP1*, *STK3*, *EXT1*, and *GRIP1* (Figure 1). These genes are functionally implicated in reproductive processes, including follicular development and maturation, cellular proliferation and apoptosis, and litter size regulation (41–43) (Table 2). Notably, a cluster of SNPs was identified within the *MMP16* gene on chromosome 9, suggesting its potential role as a key genetic determinant of reproductive performance.

**Table 2:** The information of candidate genes identified by GWAS.

Gene	ID	OAR	Range (bp)	Location	Function
*MMP16*	101,116,410	9	87,307,873–87,696,184	87,624,215 87,630,812 87,624,693 87,630,811 87,623,829 87,624,472	Promoting ovulation; the proliferation, apoptosis and steroid secretion of GCs
*STK32B*	101,103,155	6	104,404,659–104,788,232	104,669,145 104,670,564 104,656,289 104,670,619 104,663,303 104,656,837 104,662,010 104,662,009 104,672,467	Premature labor
*GRIP1*	101,115,022	3	152,867,915–153,639,173	152,876,601	Regulating estrogen affects estrus
*SLIT2*	100,125,611	6	40,397,721–40,801,150	40,538,302 40,540,204	Regulating litter size
*COL14A1*	101,120,758	9	94,433,640–94,658,871	94,650,960 94,658,230 94,646,044	Regulating ECM affects follicular development
*EHHADH*	101,117,635	1	202,654,806–202,722,993	202,691,464	Reproductive traits
*PKN1*	101,118,101	5	9,624,203–9,652,957	9,647,107	Embryonic development and cell proliferation
*BMP1*	101,122,334	2	43,399,903–43,445,910	43,427,482 43,433,712	Regulating ECM affects follicular development
*EGFR*	780,479	19	880,958–1,095,437	990,141	Regulate the proliferation, apoptosis and steroid secretion of GCs Promote follicular development
*BCNR3*	101,106,808	1	70,739,003–70,961,221	70,756,567	Embryo development
*MALRD1*	114,117,539	13	19,975,314–20,576,159	20,059,486	Gestation period
*ISLR*	101,110,878	18	32,166,790–32,169,937	32,167,770	Embryo development and miscarriage
CCS*E*R1	101,117,333	6	33,696,800–35,188,219	35,107,183	Reproductive traits

### 3.3 Expression of MMP16 gene in different tissues and localization in ovary

**Figure 2:**
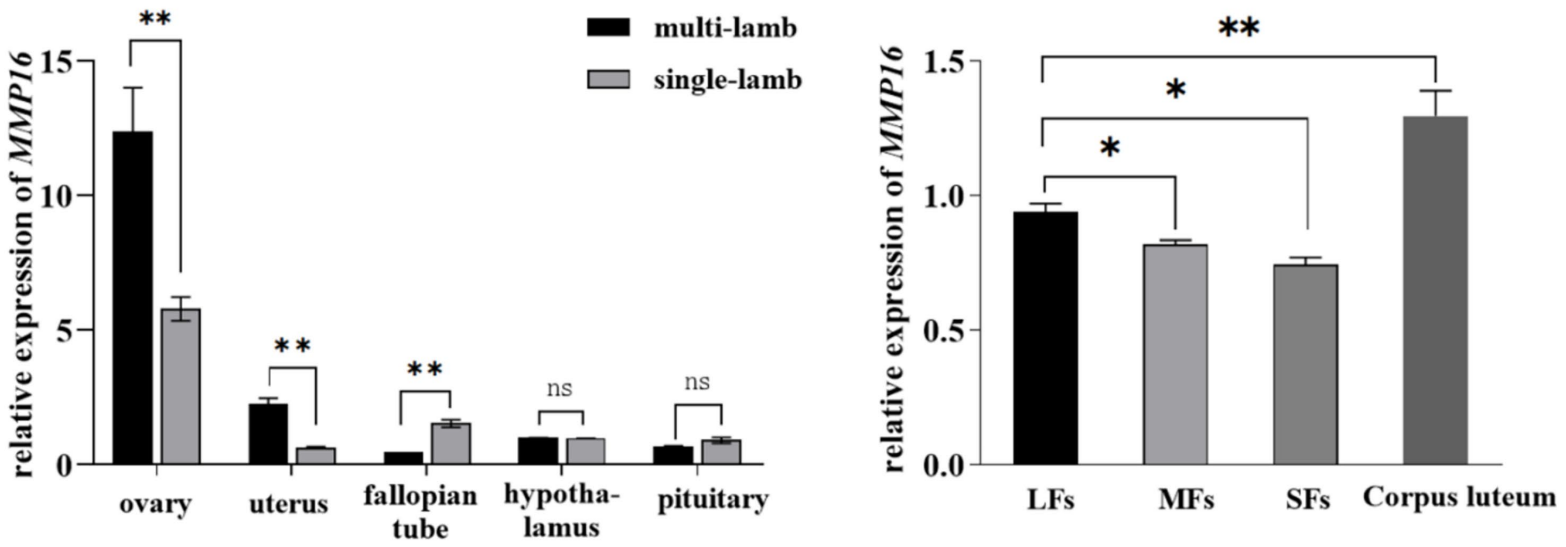
Expression of *MMP16* in different tissues and follicles. **(a)** Relative expression of *MMP16* in ovary, uterus, fallopian tube, hypothalamus and pituitary of single-lamb and multi-lamb sheep. **(b)** The relative expression levels of *MMP16* in corpus luteum, large (LFs), medium (MFs), and small (SFs) follicles. *denotes significant, **denotes highly significant.

To investigate the role of *MMP16* in sheep reproduction, we used qPCR and immunohistochemistry to analyze the expression of *MMP16* in different tissues and the localization in sheep ovary. We examined the relative expressions of *MMP16* in ovary, uterus, fallopian tube, hypothalamus and pituitary of single-lamb and multi-lamb (Figure 2). The results showed that *MMP16* gene expressed in all five tissues of both the single-lamb and multi-lamb sheep, with the highest expression in ovary (Figure 2a). The expression of *MMP16* in the ovary and uterus of the multi-lamb sheep was significantly higher than that in the single-lamb sheep (*p* < 0.01), but the expression in the fallopian tube was significantly lower than that in the single-lamb sheep (*p* < 0.01) (Figure 2b). The *MMP16* exhibited stage-specific expression patterns in ovarian follicles, with transcript levels progressively increasing throughout folliculogenesis. The highest expression was observed in the corpus luteum, while large follicles demonstrated significantly elevated *MMP16* expression compared to medium and small follicles (*p* < 0.05).

**Figure 3:**
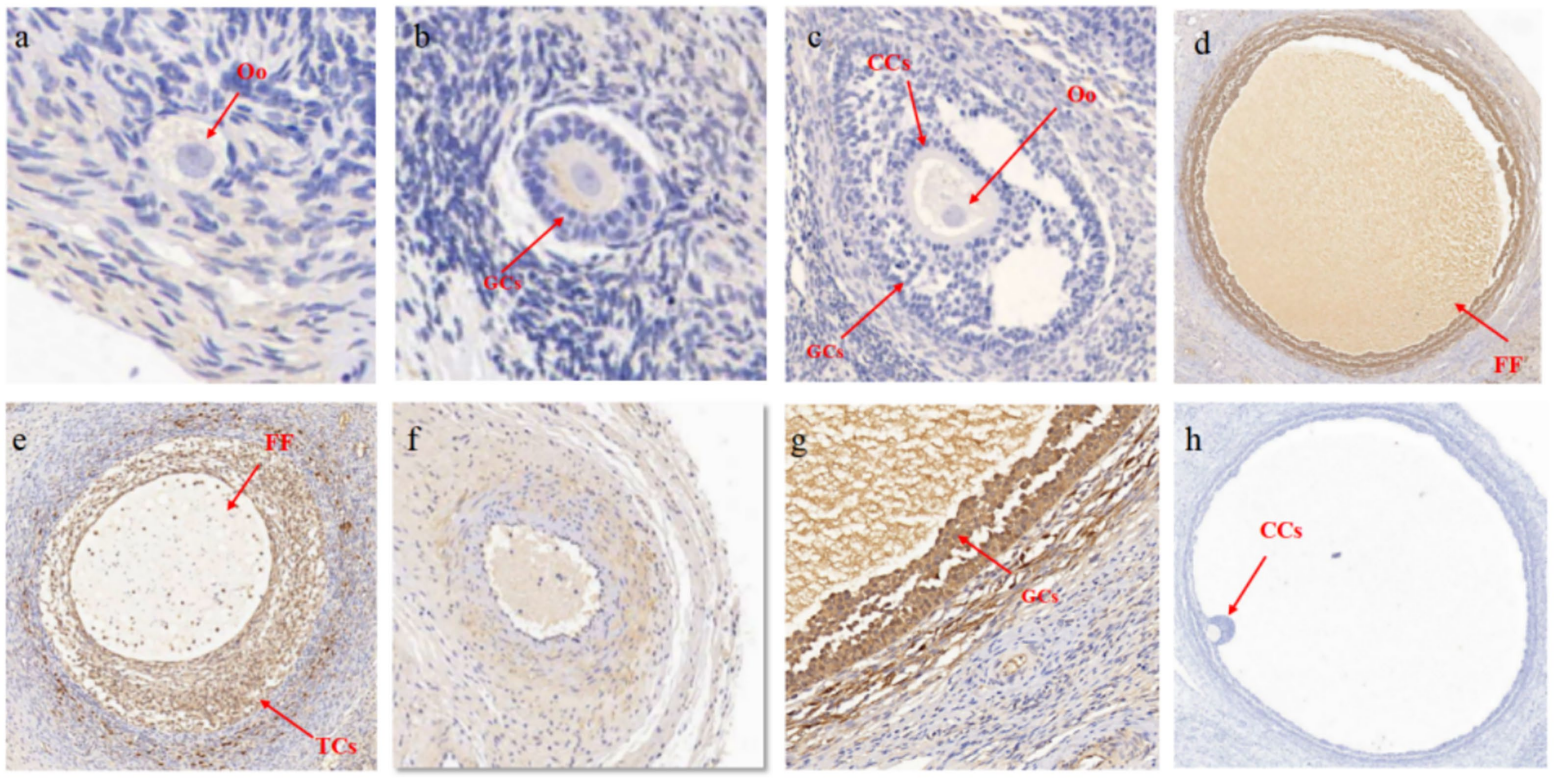
Localization of MMP16 in ovaries. **(a)** Primordial follicles, 50 μM. **(b)** Primary follicle, 100 μM. **(c)** Secondary follicles, 200 μM. **(d)** Mature follicles, 500 μM. **(e)** Large coelomic follicles, 500 μM. **(f)** corpus luteum, 200 μM. **(g)** GCs, 100 μM. **(h)** Negative control, 500 μM. CCs, cumulus cells; Oo, oocyte; TCs, theca cells; FF, follicular fluid.

The results of IHC also demonstrated stage-specific localization of MMP16 during ovine folliculogenesis (Figure 3). MMP16 was located at different developmental stages of follicles, fills the entire follicular fluid in mature follicles, and was located in oocyte, GCs, cumulus cells, theca cells and corpus luteum. These results suggest that the *MMP16* gene plays a role in ovine ovarian follicle maturation, ovulation and luteal formation.

### 3.4 Validation of MMP16 overexpression and interference efficiency in GCs

**Figure 4:**
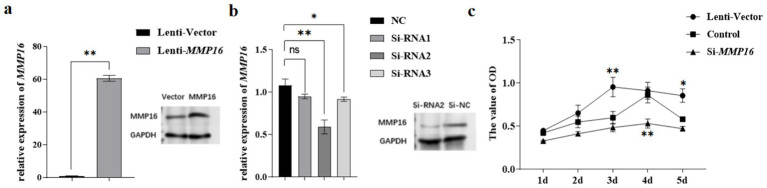
Validation of *MMP16* overexpression and interference efficiency in GCs. **(a)** The overexpression efficiency of *MMP16* in GCs was detected by qPCR and western blot. **(b)** The interference efficiency of *MMP16* in GCs was detected by qPCR and western blot. **(c)** CCK-8 measured cell viability after overexpression/interference with *MMP16*. *denotes significant, **denotes highly significant.

The qPCR and western blot analyses revealed that transfection with the lentiviral plasmid significantly upregulated *MMP16* mRNA expression levels compared to the vector (*p* < 0.01), while concurrently demonstrating a marked increase in MMP16 protein expression (Figure 4a). After transfection of the three groups of si-RNA, the results showed that compared with the NC group, si-RNA2 significantly down-regulated the expression of *MMP16* gene in GCs (*p* < 0.01), and si-RNA3 significantly down-regulated the expression of *MMP16* gene in GCs (*p* < 0.05). There was no significant change in *MMP16* gene expression in si-RNA1 (*p* > 0.05) (Figure 4b). Western blot analysis demonstrated that the expression level of MMP16 protein was significantly down-regulated in the si-RNA2. Based on these findings, si-RNA2 was selected for subsequent functional studies investigating GCs.

### 3.5 The effects of MMP16 on the viability and proliferation of GCs

To assess the effects of *MMP16* on the viability and proliferation of GCs, we employed qRT-PCR, CCK-8 assay, EdU, and TUNEL analysis. The CCK-8 assay demonstrated that the *MMP16* increased the viability of GCs on day 3 compared to the vector group (*p* < 0.01), while interfering with the expression of *MMP16* yielded opposite results (Figure 4c). The Si-RNA2 interfered with the expression of *MMP16* in GCs induced progressive suppression of activity, with culminating in statistically significant decreased on day 4 (*p* < 0.01).

**Figure 5:**
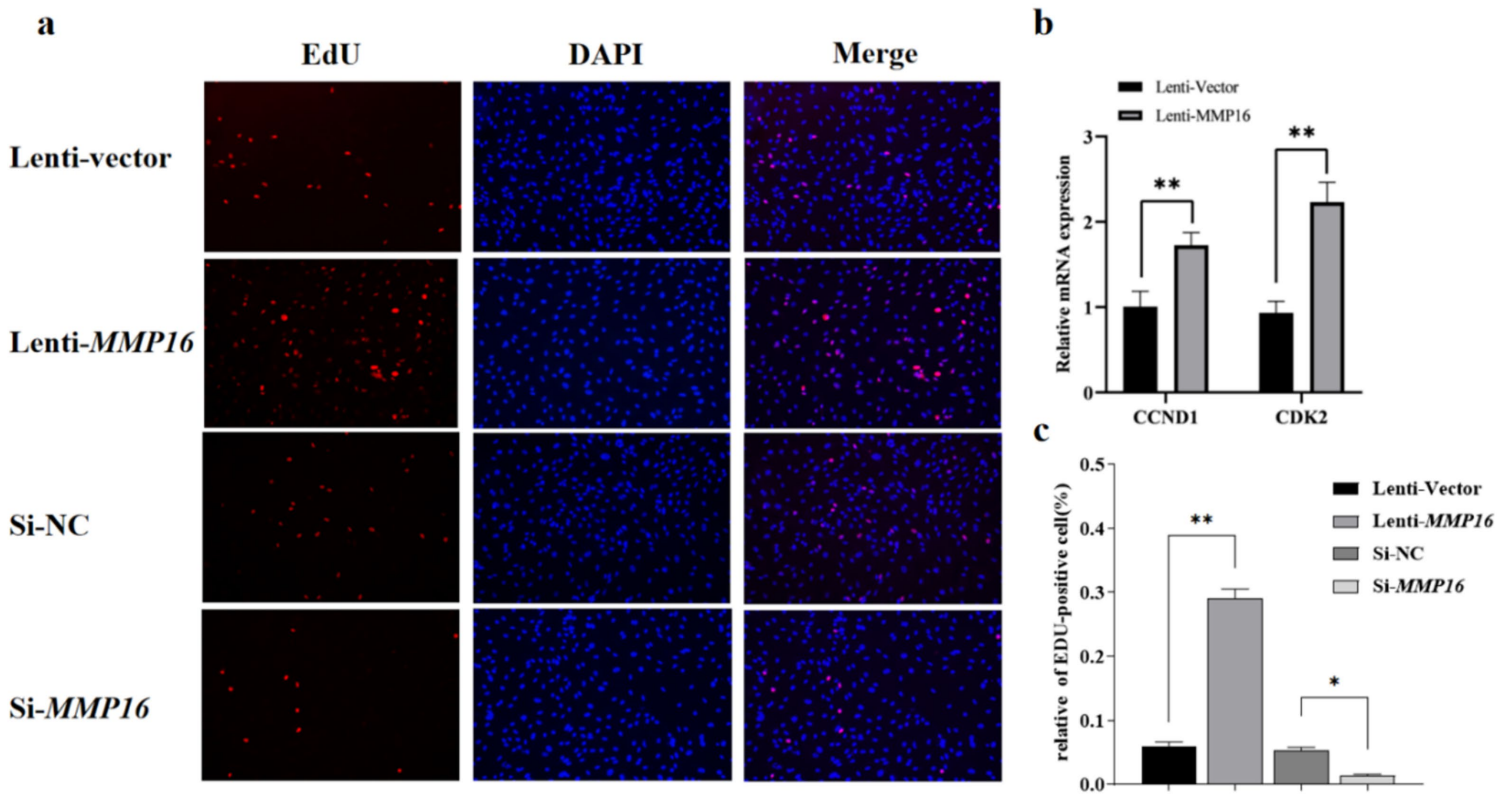
Effects of overexpression and interference of *MMP16* on the proliferation ability of sheep GCs. **(a,c)** Proliferation of GCs was assayed using EdU assays after transfection with *MMP16*. **(b)** The relative expression levels of *CCND1* and *CDK2* after transfection with *MMP16*. *denotes significant, **denotes highly significant.

**Figure 6:**
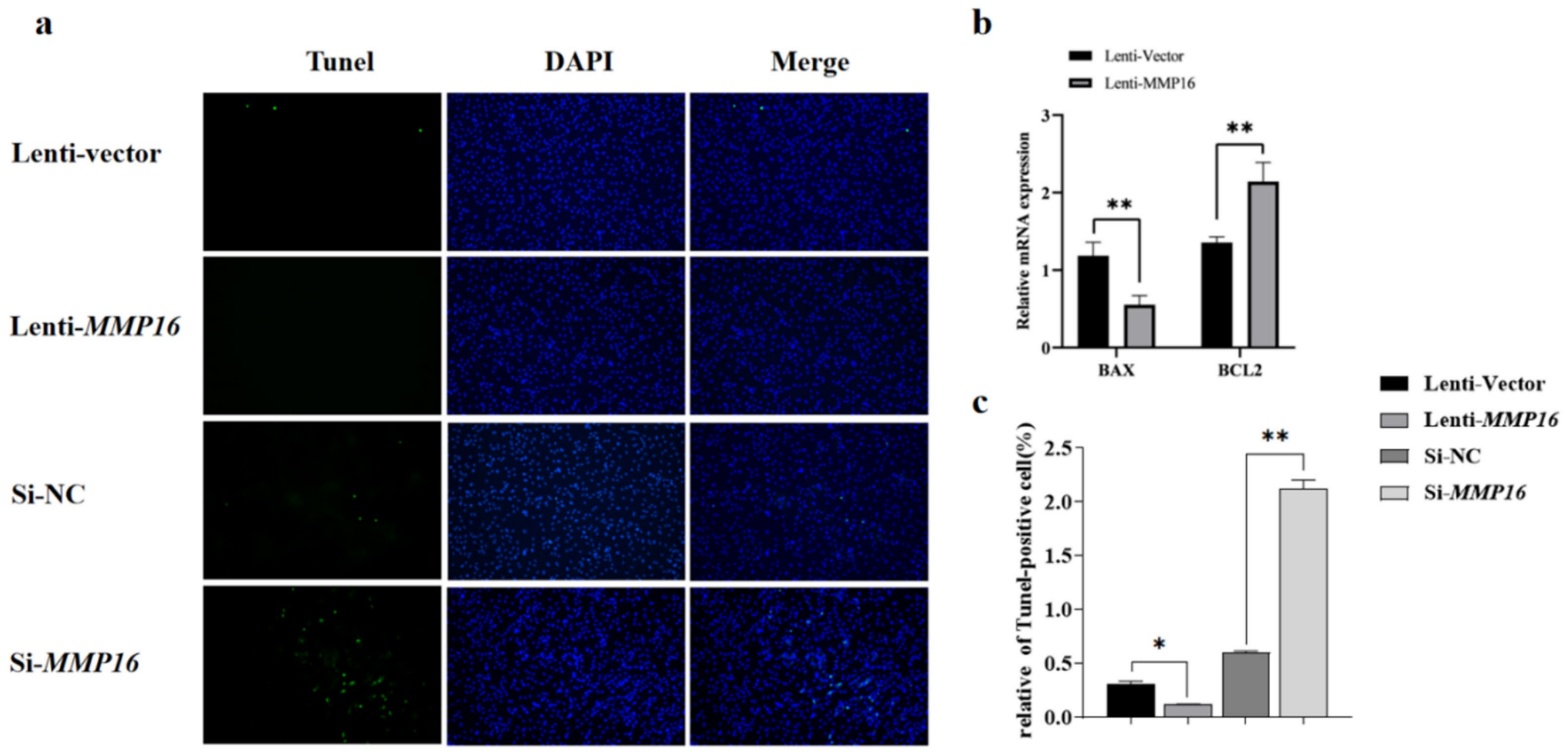
Effects of overexpression and interference of *MMP16* on the apoptosis ability of sheep GCs. **(a,c)** Apoptosis of GCs was assayed using TUNEL after transfection with *MMP16*. **(b)** The relative expression levels of *BAX* and *BCL2* after transfection with *MMP16*. *denotes significant, **denotes highly significant.

Furthermore, the EdU analysis indicated that the *MMP16* significantly promoted the proliferation of GCs compared to the vector group, whereas interfering with the expression of *MMP16* had the opposite effect. Overall, these results suggested that *MMP16* could increase the viability and promote the proliferation of GCs. TUNEL analysis was used to detect the effect of *MMP16* on the apoptosis of GCs (Figure 5). The results showed that overexpression of *MMP16* inhibited the apoptosis of GCs (*p* < 0.05), while interference with *MMP16* promoted the apoptosis of GCs (*p* < 0.01). In order to further explore the effects of *MMP16* on proliferation and apoptosis of GCs, qPCR was used to detect the expressions of proliferation-related genes *CCND1* and *CDK2*, apoptosation-related genes *BAX* and *BCL2*. The qPCR results revealed that *MMP16* overexpression significantly increased the expression of *CCND1*, *CDK2*, and *BCL2* gene (*p* < 0.01), and significantly reduced the expression of *BAX* (*p* < 0.01) (Figure 5b and Figure 6b). Apoptosis of GCs was assayed using TUNEL after transfection with MMP16 (Figures 6a, c).

### 3.6 Impact of MMP16 overexpression and interference on the steroid hormone levels of GCs and the expression of associated genes

**Figure 7:**
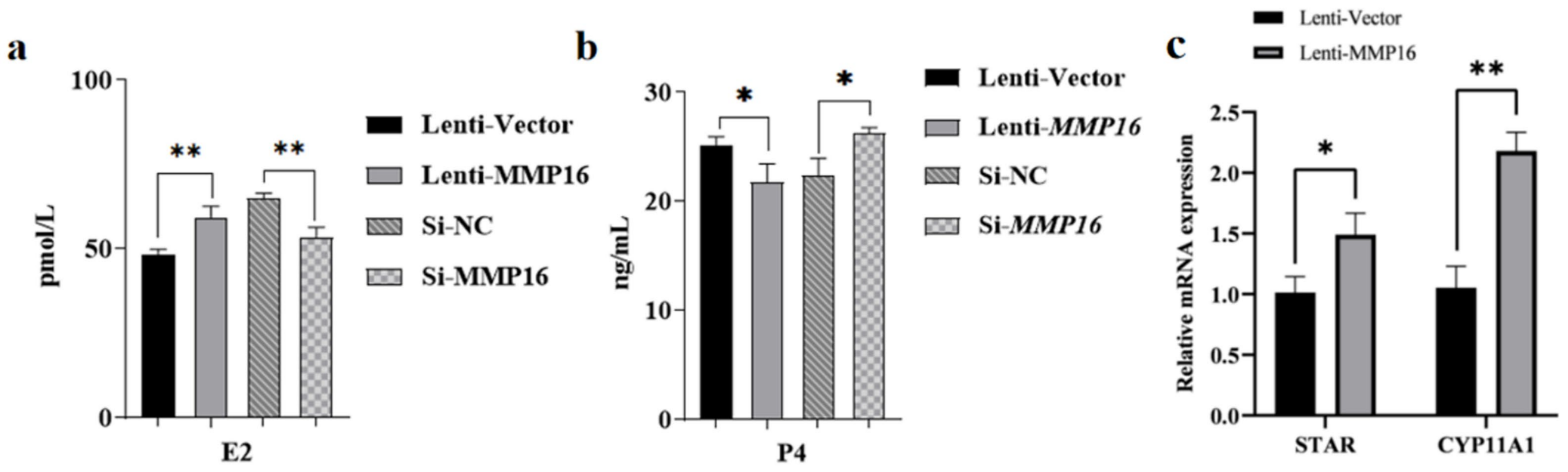
The effects of MMP16 overexpression and interference on steroid hormones and related genes. **(a)** The concentration of E2 after transfection with *MMP16*. **(b)** The concentration of P4 after transfection with *MMP16*. **(c)** The relative expression levels of *STAR* and *CYP11A1* after transfection with *MMP16*. *denotes significant, **denotes highly significant.

We examined the impact of *MMP16* overexpression and interference after 48 h on the expression of steroid synthesis-related genes and hormone levels (Figure 7). The influence of overexpression of *MMP16* on E2 concentration was significantly higher than that of vector (*p* < 0.05) (Figure 7a), and on P4 concentration was significantly lower than that of vector (*p* < 0.05) (Figure 7b), while interference of *MMP16* had the opposite effect. The mRNA expressions level of *STAR* and *CYP11A1*, key enzymes in steroid hormone synthesis pathway, significantly increased compared with the vector group (*p* < 0.05 or *p* < 0.01) (Figure 7c). These results suggest that *MMP16* may regulate the expression of steroid synthesis-related genes.

### 3.7 Transcriptome sequencing analysis

**Figure 8:**
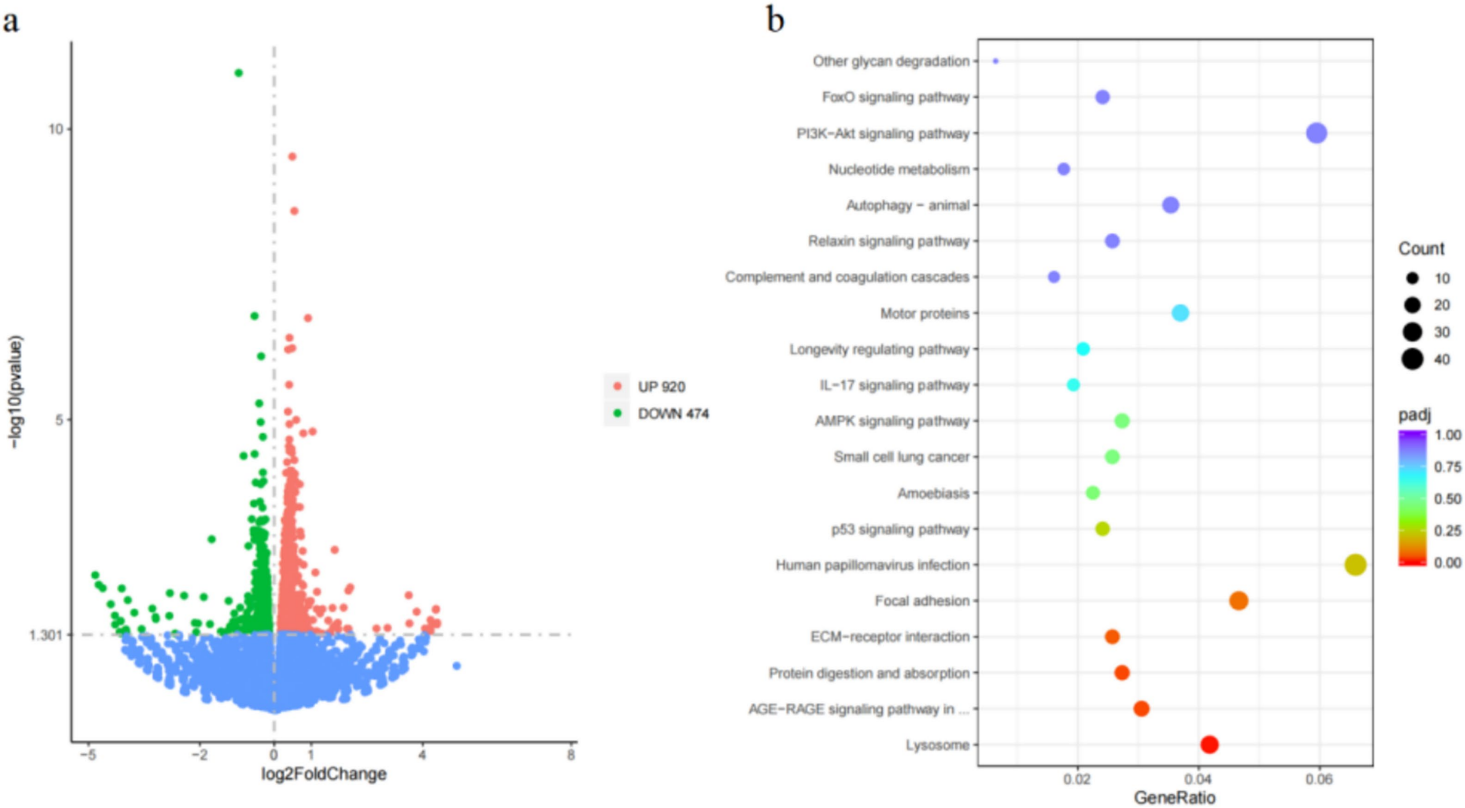
Differential gene identification and enrichment analysis results. **(a)** Differential gene volcano map after *MMP16* overexpression in sheep ovarian granulosa cells. **(b)** Bubble map of KEGG enrichment analysis.

To investigate further regulatory mechanisms of *MMP16*, we performed transcriptome sequencing to identify potential signaling pathways after *MMP16* overexpression (Figure 8). The sequencing results showed that a total of 296,607,618 raw reads were obtained. After quality control, a total of 292,873,450 clean reads were obtained. The volcano plot depicting DEGs revealed that the transcript levels of 920 genes were significantly up-regulated (Padj < 0.05), while 474 genes exhibited significant down-regulation (Padj < 0.05) (Figure 8a). Subsequent KEGG analysis of the DEGs unveiled that these genes were notably enriched in pathways associated with the ECM-receptor interaction as well as the PI3K-Akt signaling pathway (Figure 8b). Furthermore, transcriptome sequencing detected the expressions of *CCND1*, *CDK2*, *BCL2*, *STAR*, and *CYP11A1* gene significantly increased, and the expression of *BAX* significantly decreased, which was consistent with our qPCR results.

### 3.8 The expression of related genes in the PI3K-AKT signaling pathway

**Figure 9:**
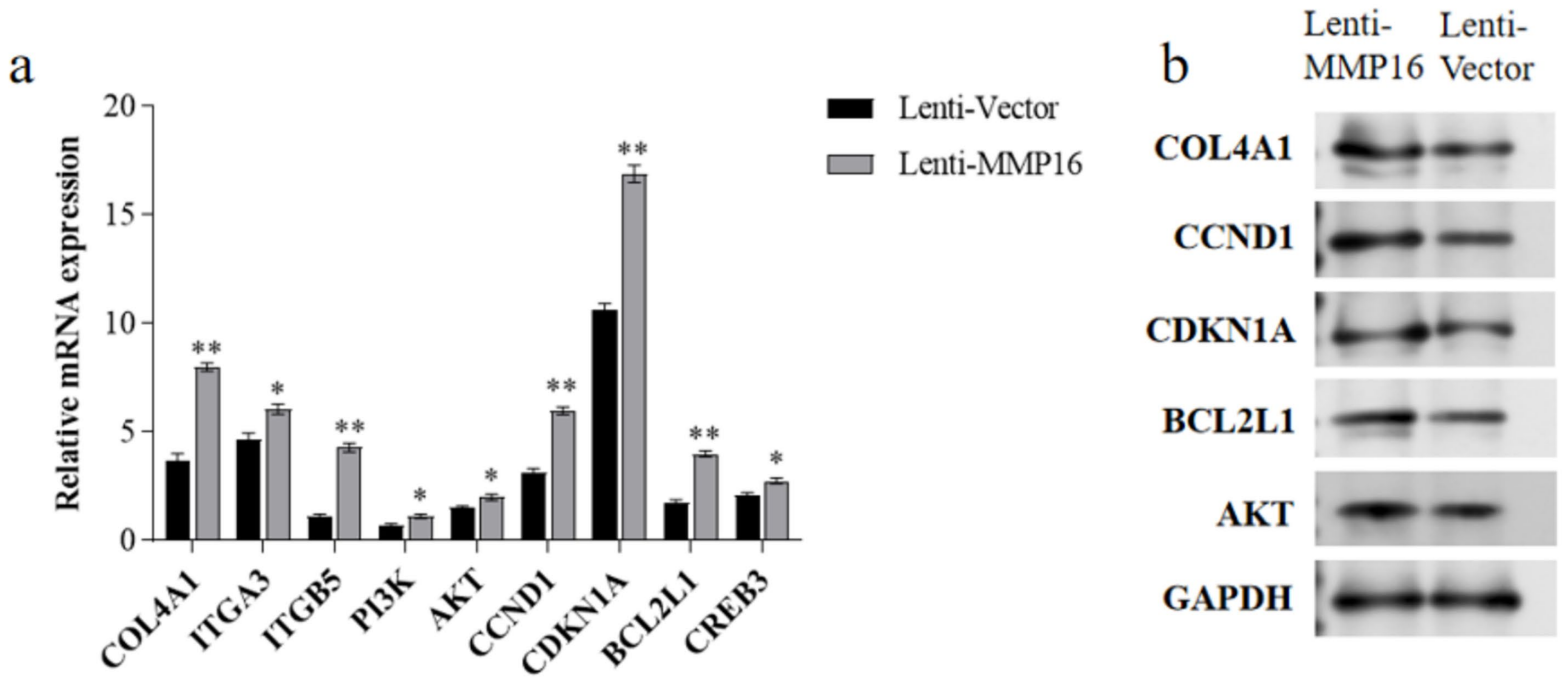
The expressions of related genes in the PI3K-AKT signaling pathway. **(a)** The relative mRNA expressions of related genes in the PI3K-AKT signaling pathway. **(b)** The protein expressions of related genes in the PI3K-AKT signaling pathway. *denotes significant, **denotes highly significant.

The mRNA expression levels of *COL4A1*, *ITGA3*, *ITGB5*, *PI3K*, *AKT*, *CCND1*, *CDKN1A*, *BCL2L1* and *CREB3* in the PI3K-AKT signaling pathway were detected by qPCR and western blot (Figure 9). The results showed that the expression levels of these related genes significantly increased after overexpression of *MMP16* (Figure 9a), which was consistent with the result of protein expression (Figure 9b).

## 4 Discussion

Genetic improvement of litter size in livestock represents a promising strategy for enhancing production efficiency. Through genome-wide association study, we identified key candidate genes associated with litter size traits in Lop sheep, and further elucidated the molecular regulatory mechanism of *MMP16* in GCs. These findings provide novel insights into the genetic architecture underlying prolificacy in sheep and offer valuable references for understanding mammalian reproductive efficiency.

Reproductive processes are inherently complex, with quantitative traits such as ovulation rate and litter size being governed by the cumulative effects of major-effect mutations and minor-effect loci within a polygenic regulatory framework. *BMP1* was initially identified in osseous extracts demonstrating ossification-inducing activity at ectopic sites (44), and subsequent investigations revealed its critical role in modulating GCs proliferation and apoptosis, thereby participating in the physiological process of follicular selection (45). The estrogen receptor coactivator *GRIP1* is functionally conserved in ovarian tissues across sheep, bovine, and porcine species, and its expression level governs the transcriptional activation of estrogen-responsive genes, thereby modulating systemic hormonal responsiveness in target reproductive tissues (46). The expression of *EGFR* was identified in sheep ovarian follicles at different periods, with melatonin demonstrating modulatory effects on its expression levels, suggesting *EGFR* may exert stage-specific regulatory roles during follicular development (47, 48). *SLIT2* expressed in ovary, with its expression demonstrating luteal phase-specific upregulation (13). Notably, this spatiotemporal expression pattern was negatively regulated by human chorionic gonadotropin and cortisol, and pharmacological inhibition of the SLIT-ROBO signaling enhanced luteal cell migration capacity and reduced apoptotic rate (49). These results suggest that *SLIT* may be involved in ovarian development by controlling the migration or apoptosis of cells.

*MMP16*, a membrane-type matrix metalloproteinase (MT-MMP), critically regulates extracellular matrix (ECM) homeostasis through spatiotemporal proteolytic remodeling, positioning it as a key modulator in both physiological tissue morphogenesis and pathological conditions such as cancer metastasis and fibrosis (50–53). Within the female reproductive axis, *MMP16* serves as a pivotal modulator of folliculogenesis through dynamic ECM proteolysis. Its spatiotemporal remodeling of basement membrane components orchestrates critical transitions including follicular selection, ovulation, and corpus luteum formation, while simultaneously modulating atresia via granulosa cell apoptosis regulation (54). *MMP16* exhibits its highest expression levels in the ovary, with a progressive increase observed throughout follicular development, ultimately reaching peak expression in the corpus luteum, which implicates *MMP16* in orchestrating key reproductive processes including folliculogenesis, ovulation, and luteal formation. Comparative analysis revealed significantly higher *MMP16* expression levels in ovarian tissues of multi-lamb compared to single-lamb, suggesting its potential regulatory role in litter size through follicular development optimization. Previous study has established ECM components as predominant proteolytic substrates for collagenases and gelatinases, with GCs specific expression of these proteases being essential for follicular wall ECM degradation and subsequent ovulation facilitation through targeted matrix remodeling (55). During the ovulation period of rats and humans, *MMP16* is regulated by chorionic gonadotropin, which upregulates the proteolytic activity within the follicles, thereby driving the occurrence of follicular ovulation (56). Furthermore, some studies have also found that in mouse GCs, the increased expression of MT-MMPs and *TIMP1* lead to an increase in *MMP2* (55). Our study similarly demonstrated that *MMP16* overexpression significantly upregulated *MMP2* expression based on transcriptome sequencing data. In follicles, *MMP2* and *MMP9* proteins predominantly localized to follicular tissue remodeling sites, with their mRNA and protein levels positively correlating with follicular diameter (57). Synthesized and secreted by GCs and theca cells of small antral follicles, these proteases are hypothesized to participate in dominant follicle selection, given that follicular dominance is established during the antral stage (58). Falkowski unveiled an additional regulatory mechanism where in the MMP14-TIMP1 complex activates theca/granulosa-derived *MMP2* upon migratory cumulus-oocyte complex, triggering rapid apical follicular rupture and oocyte extrusion (59).

Furthermore, we observed that *MMP16* overexpression was significantly associated with enhanced PI3K-AKT pathway activity, suggesting its potential to promote ovine follicular maturation and ovulation via activation of this key intracellular signaling cascade. The PI3K-AKT pathway, a well-characterized signaling pathway, plays pivotal roles in diverse physiological processes including cellular proliferation, differentiation, apoptosis, and metabolic regulation. Recent studies have established its essential involvement throughout folliculogenesis, spanning primordial follicle recruitment, GCs proliferation, corpus luteum survival, and oocyte maturation (60). Within the PI3K-AKT pathway, coordinated upregulation of the cell cycle regulator *CCND1* and downregulation of *CDKN1A* collectively drive GCs proliferation, a finding corroborated by EdU assay results. *CYP11A1*, a key enzyme regulating steroid hormone biosynthesis in GCs, modulates follicular development and ovulation through hormonal dynamics (61). Notably, our study demonstrates that *MMP16* significantly upregulates *CYP11A1* expression, implicating its functional role in potentiating GCs steroidogenic activity. Concurrently, AMPK, a crucial intracellular energy sensor, maintains cellular energy homeostasis and regulates metabolic processes. Within the reproductive system, AMPK activity alterations exert profound implications on follicular development and ovulation. Specifically, FSH-activated PI3K/AKT and AMPK pathways play critical roles in orchestrating GCs mitotic progression and cell cycle regulation (62). *MMP16* overexpression effectively suppresses AMPK phosphorylation, thereby diminishing its activity, which likely facilitates follicular maturation and ovulation (63). In summary, by inhibiting AMPK activity and activating the PI3K-AKT pathway, *MMP16* promotes GCs proliferation, suppresses apoptosis, and regulates steroid hormone synthesis. These integrated effects collectively enhance ovulation efficiency and litter size.

## 5 Conclusion

This study integrated population genetics analysis and functional validation to elucidate the critical role of the *MMP16* gene in establishing high fecundity traits in Luobo sheep. Genome-wide association study identified *MMP16* as significantly associated with litter size variation. Tissue-specific expression profiling demonstrated predominant ovarian expression of *MMP16*, with peak levels localized to the corpus luteum and mature follicles. IHC analysis further confirmed MMP16 protein expression in granulosa and theca cells, indicating its involvement in follicular development, maturation and ovulation regulation. Functional investigations revealed that *MMP16* overexpression enhances GCs proliferation, suppresses apoptosis, and modulates ECM remodeling through PI3K-AKT pathway activation concurrent with AMPK phosphorylation inhibition. Coordinated upregulation of steroidogenic genes further substantiated its role in follicular maturation. These findings provide both a theoretical breakthrough in understanding the genetic basis of ovine hyperprolificacy and a scientific foundation for precision breeding strategies to enhance sheep production efficiency.

## Data Availability

The datasets analyzed during the current study are available, China National Center for Bioinformation/Beijing Institute of Genomics, Chinese Academy of Sciences repository, accession number: GVM000916.

## Footnotes

^1^ https://www.bioinformatics.babraham.ac.uk/
